# COX-2/sEH Dual Inhibitor PTUPB Attenuates Epithelial-Mesenchymal Transformation of Alveolar Epithelial Cells *via* Nrf2-Mediated Inhibition of TGF-*β*1/Smad Signaling

**DOI:** 10.1155/2022/5759626

**Published:** 2022-04-25

**Authors:** Chen-Yu Zhang, Xin-Xin Guan, Zhuo-Hui Song, Hui-Ling Jiang, Yu-Biao Liu, Ping Chen, Jia-Xi Duan, Yong Zhou

**Affiliations:** ^1^Department of Physiology, School of Basic Medicine Science, Central South University, Changsha, Hunan 410078, China; ^2^Department of Pulmonary and Critical Care Medicine, The Second Xiangya Hospital, Central South University, Changsha, Hunan 410078, China; ^3^Department of Physiology, Changzhi Medical College, Changzhi, Shanxi 046000, China; ^4^Research Unit of Respiratory Disease, Central South University, Changsha, Hunan 410011, China

## Abstract

**Background:**

Arachidonic acid (ARA) metabolites are involved in the pathogenesis of epithelial-mesenchymal transformation (EMT). However, the role of ARA metabolism in the progression of EMT during pulmonary fibrosis (PF) has not been fully elucidated. The purpose of this study was to investigate the role of cytochrome P450 oxidase (CYP)/soluble epoxide hydrolase (sEH) and cyclooxygenase-2 (COX-2) metabolic disorders of ARA in EMT during PF.

**Methods:**

A signal intratracheal injection of bleomycin (BLM) was given to induce PF in C57BL/6 J mice. A COX-2/sEH dual inhibitor PTUPB was used to establish the function of CYPs/COX-2 dysregulation to EMT in PF mice. *In vitro* experiments, murine alveolar epithelial cells (MLE12) and human alveolar epithelial cells (A549) were used to explore the roles and mechanisms of PTUPB on transforming growth factor (TGF)-*β*1-induced EMT.

**Results:**

PTUPB treatment reversed the increase of mesenchymal marker molecule *α*-smooth muscle actin (*α*-SMA) and the loss of epithelial marker molecule E-cadherin in lung tissue of PF mice. *In vitro*, COX-2 and sEH protein levels were increased in TGF-*β*1-treated alveolar epithelial cells (AECs). PTUPB decreased the expression of *α*-SMA and restored the expression of E-cadherin in TGF-*β*1-treated AECs, accompanied by reduced migration and collagen synthesis. Moreover, PTUPB attenuated TGF-*β*1-Smad2/3 pathway activation in AECs *via* Nrf2 antioxidant cascade.

**Conclusion:**

PTUPB inhibits EMT in AECs *via* Nrf2-mediated inhibition of the TGF-*β*1-Smad2/3 pathway, which holds great promise for the clinical treatment of PF.

## 1. Introduction

Pulmonary fibrosis (PF) is a prototype of chronic, progressive, and fibrotic lung disease. An altered extracellular matrix replaces healthy tissue, and alveolar architecture is destroyed, which leads to decreased lung compliance, disrupted gas exchange, and ultimately respiratory failure and death [1]. Although pirfenidone and nintedanib have been authorized by the Food and Drug Administration [2], they only slow down lung function decline in patients with the mild and moderate disease [3]. Therefore, it is urgent to develop an effective treatment for PF.

Epithelial-mesenchymal transition (EMT) is a reversible process in which epithelial cells lose their cellular polarity and obtain migration characteristics through down-regulation of E-cadherin-mediated cell adhesion [4]. EMT is involved in wound healing, fibrosis, embryonic development, and cancer metastasis [5]. Most investigators concur that alveolar type II epithelial cells undergo EMT during PF development [6, 7]. Studies have shown that pulmonary fibroblasts are derived from various routes, of which about one-third are derived from alveolar type II epithelial cells *via* EMT [8]. Transforming growth factor (TGF)-*β*1 is the most studied and is a key EMT inducer [9]. TGF-*β*1 activates its downstream Smad signaling pathway and plays an important role in fibrosis [10]. TGF-*β*1 binds to its receptor to trigger intracellular signaling and phosphorylates Smad2 and Smad3. Phosphorylated Smad2 and Smad3 are transported to the nucleus and regulate the transcription of target genes [11]. Consequently, blocking the EMT of alveolar epithelial cells (AECs) might be a promising strategy for the treatment of PF.

Oxidative stress accelerates TGF-*β*1-mediated fiber formation by increasing hydrogen peroxide levels, protein damage, DNA degradation, and lipid peroxidation [12]. The transcription factor nuclear factor erythroid 2-related factor-2 (Nrf2) plays an important role in intracellular antioxidant responses. Activated Nrf2 is transported to the nucleus to promote the transcription of antioxidant enzymes [13]. Nrf2 balances not only oxidative stress but also has negative effects on TGF-*β*1-mediated profibrotic signal transduction [14, 15]. Previous studies have shown that Nrf2 plays an important role in preventing lung inflammation and fibrosis [16, 17]. These results indicate that strategies targeting Nrf2 have antipulmonary fibrosis potential.

Epoxyeicosatrienoic acids (EETs), leukotrienes (LTs), and prostaglandins (PGs) are derived from arachidonic acid (ARA) with cytochrome P450 oxidase (CYP), lipoxygenase (LOX), and cyclooxygenase (COX) pathways, respectively [18]. ARA metabolites play multiple roles in almost diseases. A previous study found that the knockdown of COX-2 can reduce TGF-*β*1-induced EMT, indicating that the increased expression of COX-2 is involved in the process of EMT [19]. The up-regulated expression of COX-2 stimulates the production of TGF-*β*, which is inhibited by NS-398, a selective inhibitor of COX-2 [20, 21]. The activation of a TGF-*β*1/Smad3 signaling pathway is modulated by an up-regulated expression of COX-2 in benzalkonium chloride-induced subconjunctival fibrosis [10]. In addition, COX-2 has been shown to promote cancer initiation and progression through pleiotropic functions, including EMT induction *via* its predominant product, PGE2, which binds to the cognate receptor EP2 [22]. These studies suggest that the COX-2 metabolism of ARA promotes the process of EMT. ARA metabolism generates EETs *via* the CYP2C/2J metabolic pathway [23]. EETs have various biological activities such as vasodilators, anti-inflammation, and anti-fibrosis [24–28]. We have reported that blockade of EETs degradation attenuates murine PF [28]. Besides, EETs inhibit EMT in unilateral ureteral obstruction (UUO) mice by decreasing renal Snail1 and Zinc-finger E-box binding (ZEB) expression [29]. However, EETs are rapidly metabolized by soluble epoxide hydrolase (sEH) [24].

Our previous study suggested that the expressions of sEH and COX-2 are significantly increased in the lungs of PF mice induced by bleomycin (BLM) [30]. A compound that concurrently inhibits both COX-2 and sEH is called 4-(5-phenyl-3-{3-[3-(4-trifluoromethylphenyl)-ureido]-propyl}-pyrazol-1-yl)-benzenesulfonamide (PTUPB), which prevents the release of PGs and increases the blood levels of EETs [31]. PTUPB is more potent in suppressing inflammatory pain and tumor growth than celecoxib, t-AUCB (an inhibitor of sEH), or the combination of celecoxib and t-AUCB [31, 32]. We have shown that PTUPB can alleviate acute lung injury [33], nonalcoholic fatty liver disease [34], and sepsis [35] in mice. What is more, we have found that PTUPB significantly attenuates BLM-induced PF in mice [30]. However, it is not clear whether PTUPB can inhibit EMT. Therefore, the present study aimed to investigate the effects of PTUPB on TGF-*β*1-mediated pulmonary EMT.

## 2. Materials and Methods

### 2.1. Animal

C57BL/6J mice (adult male, 6-8 weeks) were obtained from Hunan SJA Laboratory Animal Co., Ltd. (Hunan, China). Mice were placed in specific pathogen-free conditions for a 12 h day-night cycle. Mice have free access to food and water.

### 2.2. Murine Model of PF and Treatment

Mice were randomly divided into the control group, PTUPB group, BLM group, and BLM+PTUPB group. For PF induction, mice received an intratracheal injection of BLM (1.5 mg/kg; Nippon Kayaku, Tokyo, Japan) dissolved in 50 *μ*L saline. At the same time, mice in the control and PTUPB groups received 50 *μ*L saline intratracheally. Mice in the PTUPB group and BLM+PTUPB group were subcutaneously injected with PTUPB (5 mg/kg/d) dissolved in PEG400 from day 7 to day 21 after BLM injection. PTUPB was given by Bruce D. Hammock at UC Davis Comprehensive Cancer Center, University of California [31]. PEG400 was subcutaneously injected for the control and BLM groups. Twenty-one days after the BLM injection, the mice were sacrificed. All surgeries were performed under anesthesia.

### 2.3. Pulmonary Histopathology Analysis

The left lung tissue was placed in a tube filled with 4% paraformaldehyde (Servicebio, Wuhan, China, G1101), followed by conventional paraffin embedding. Paraffin-embedded sections were made. Hematoxylin-eosin staining (HE) was used to observe the morphological changes in lung tissue of mice, and Masson staining was used to observe the collagen deposition. The pictures were detected by a microscope (Motic, BA410E, Motic China group CO., LTD. China) equipped with Motic images plus 3.0 (Motic, Motic China group CO., LTD. China). The image was magnified at 200 ×, with a resolution of 683 × 705, horizontal and vertical resolutions of 96dpi, and a bit depth of 24.

### 2.4. Immunofluorescent Staining

The lung tissue sections were dewaxed and hydrated. EDTA buffer was used for antigen repair under high temperature and pressure conditions. 3% H_2_O_2_ was dropped on the sample for 10 min to achieve the purpose of removing endogenous peroxidase (the cells were washed with PBS three times and fixed with 4% paraformaldehyde for 15 minutes. After washing with PBS three times, the cells were permeated with 0.3% Triton X-100 for 15 minutes). The sections or cells were incubated in 5% albumin bovine V (BSA; Solarbio, Beijing, China, A8020) for 30 min and then incubated with *α*-SMA antibody (1 : 200; Abcam, Cambridge, MA, USA), E-cadherin antibody (1 : 200; Cell Signaling Technology, USA), Smad2 antibody (1 : 200; CST), Smad3 antibody (1 : 200; Abcam), or Nrf2 antibody (1 : 300; CST) in 5% BSA overnight at 4°C. The next day, tissue sections were rewarmed at 37°C for half an hour and then incubated with a FITC-conjugated goat antirabbit antibody (1 : 2000; Abcam). The nuclei were counterstained with DAPI (Invitrogen, Carlsbad, CA, USA). The coverslips were mounted in 90% glycerol in PBS. The fluorescence was detected by a fluorescence microscope (Motic, BA410E, Motic China group Co., Ltd., China) equipped with Motic images plus 3.0 (Motic, Motic China group Co., Ltd., China). The same field was photographed for green fluorescence (EX: AT480/30×, BS: AT505DC, EM: AT515lp) and DAPI (EX: AT375/28×, BS: AT415DC, EM: AT460/50 m), and then, the fields were superimposed using Image J software. The image was magnified at 200 ×, with a resolution of 1920 × 1440, horizontal and vertical resolution of 72 dpi, and a bit depth of 24.

### 2.5. Cell Culture and Treatment

Cells were cultured in an incubator at 37°C with 5% CO_2_. The A549 and MLE12 cell lines were purchased from ATCC. The immortalized human alveolar epithelial cells A549 were cultured in RPMI 1640 (Gibco, Grand Island, NY, USA) supplemented with 10% bovine calf serum (Sigma-Aldrich, St. Louis, MO, USA). Murine alveolar epithelial cells MLE-12 cells were cultured in DMEM F-12 (Gibco) supplemented with 2% bovine calf serum (Sigma-Aldrich), 1% penicillin and streptomycin (Solarbio), 1% 100 × ITS-G (insulin-transferrin-selenium,Solarbio), 10 nM hydrocortisone (Solarbio), and 10 nM *β*-estradiol (Solarbio).

To estimate the effect of PTUPB on TGF-*β*1 (10 ng/mL)-challenged AECs, a series of concentrations of PTUPB (0.1, 1, and 10 *μ*M) were added 1 h before TGF-*β*1 stimulation. To evaluate the role of Nrf2 in PTUPB-inhibited EMT, ML385 (Cat. No.: HY-100523, MCE) was added to inhibit Nrf2 1 h before the TGF-*β*1 stimulation.

### 2.6. Scratch Wound Healing Assay

A549 cells were cultured in 12-well plates with 2% bovine calf serum. Assigned areas of the cell surface were scratched with a 200-*μ*L tip and washed with phosphate buffer solution three times [36]. Cells were pretreated with PTUPB (1 *μ*M) for 1 h, followed by TGF-*β*1 (10 ng/mL; Novus Biologicals, Littleton, CO, USA). After 48 h of TGF-*β*1 treatment, the ability of cells to migrate to the scratch area was assessed by measuring the width of the scratch and calculating the difference from the initial width. Photographs were taken with a microscope (Nikon).

### 2.7. Cell Proliferation Assay

A549 cells were cultured in 96-well plates with 2% bovine calf serum. Cells were pretreated with PTUPB (1 *μ*M) for 1 h, followed by TGF-*β*1 (10 ng/mL). After TGF-*β*1 treatment for 48 h, 10 *μ*L Cell Counting Kit-8 solution (CCK-8, Dojindo Laboratories, Japan) was added to each well and incubated at 37°C for 1-3 h. The results were detected at 450 nm with a microplate analyzer (Thermo Fisher Scientific, Waltham, MA, USA).

### 2.8. Detection of ROS

ROS in the cells was assessed by kits following the manufacturer's instructions (Cat# ROS: E004, Jiancheng Bioengineering Institute, Nanjing, China).

### 2.9. The Quantitative Real-Time PCR Analysis

Total RNA from right middle lung tissue or cells was extracted with RNAiso Plus (Takara, Kusatsu, Japan). Total RNA (1 *μ*g) was reverse transcribed using PrimeScript RT reagent Kit (Takara). Real-time PCR was carried out to detect mRNA expression levels as described in our previous study [37]. Relative expression of genes was computed by the 2^-*ΔΔ*CT^ method according to our previous study [38]. The sequence of primers used in this study is shown in Table 1.

### 2.10. Western Blot

Protein from right lower lung tissue or cells was extracted according to our previous research [30]. A BCA Protein Assay Kit (Thermo Fisher Scientific, USA) was used to quantify protein concentrations. SDS-PAGE gel electrophoresis was performed, and the protein was transferred from the gel to polyvinylidene fluoride membranes (Millipore, Bedford, MA). The membranes were blocked with 5% BSA or skim milk. The membranes were probed with primary antibody against sEH (1 : 2000; Abcam), COX-2 (1 : 1000; Servicebio, Wuhan, China), Collagen I (1 : 1000; CST), E-cadherin (1 : 1000; CST), *α*-SMA (1 : 2000; SAB, College Park, MA, USA), Smad2 (1 : 1000; Abcam), Smad3 (1 : 1000; Abcam), p-Smad2 (1 : 1000; Abcam), p-Smad3 (1 : 1000; Abcam), Nrf2 (1 : 2000; CST), HO-1(1 : 1000; Abcam), *α*-Tubulin (1 : 5000; Servicebio), *β*-Tubulin (1 : 5000; Proteintech, Rosemont, IL, USA), or GAPDH (1 : 2000; Servicebio). The primary antibody was incubated overnight. Horseradish peroxidase-conjugated secondary antibodies (1 : 5000; CST) at room temperature for 1 h and enhanced chemiluminescence (Millipore, USA) were applied to detect protein content. Images were captured on the Chemidoc XRS (Bio-Rad) instrument. The bands were quantified using image laboratory analyzer software (Bio-Rad).

### 2.11. Statistical Analyses

All data were presented as means ± standard deviation. Statistical analysis was performed using GraphPad Prism 7 (GraphPad Software, Inc., San Diego, CA, USA). Multiple group comparisons were made using a one-way analysis of variance. Tukey's test was used as a post hoc test to make pairwise comparisons. Differences between the two groups were determined by an unpaired *t*-test. All experiments were independently repeated three times. *P* < 0.05 was considered statistically significant.

## 3. Results

### 3.1. PTUPB Reduces PF in Mice Induced by BLM

**Figure 1:**
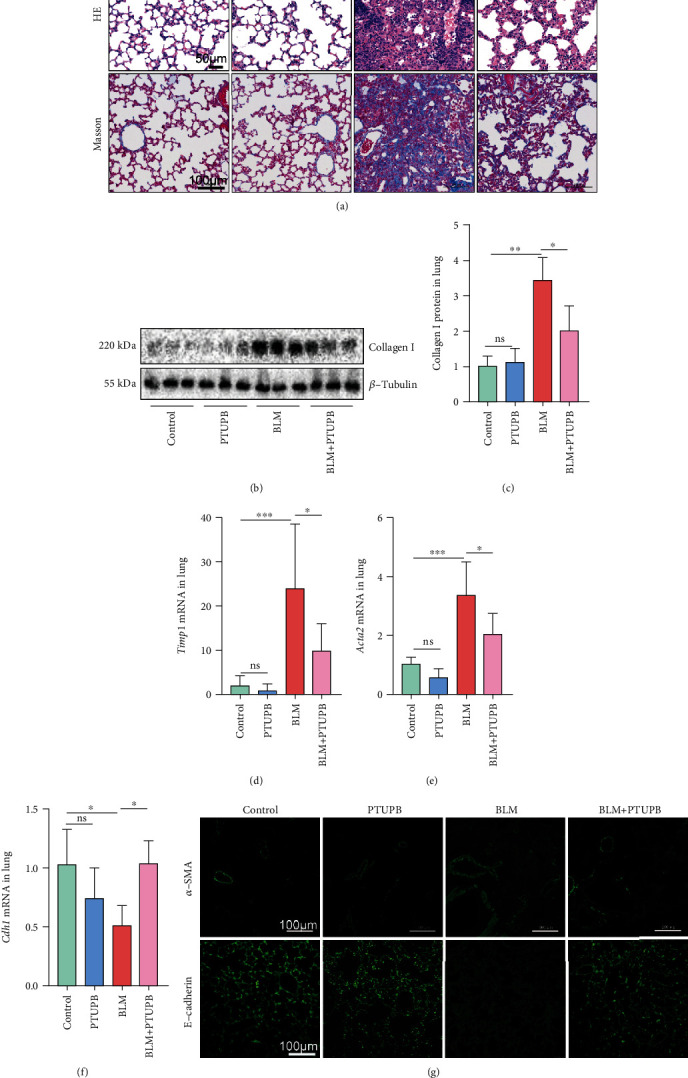
PTUPB reduces pulmonary fibrosis in mice induced by BLM. Mice received an intratracheal injection of BLM (1.5 mg/kg). Twenty-one days later, HE and Masson staining were employed to evaluate the pulmonary morphology changes and collagen disposition ((a) HE staining: bar =50 *μ*m; Masson staining: bar =100 *μ*m). The Collagen I protein expression was detected by western blotting ((b–c) *n* = 6). The mRNA expressions of *Timp1*, *Acta2*, and *Cdh1* were detected by real-time PCR ((d–f) *n* = 5 − 6). The deposition of *α*-SMA and E-cadherin was detected by immunofluorescence ((g) bar =100 *μ*m). Data are expressed as the mean ± SD. Differences among multiple groups were performed using ANOVA. Tukey's test was used as a post hoc test to make pairwise comparisons. ∗*P* < 0.05, ∗∗*P* < 0.01, and ∗∗∗*P* < 0.001.

In this study, a COX-2/sEH dual inhibitor PTUPB (5 mg/kg, s.c. once a day) was employed on the 7th day after BLM administration. HE and Masson staining results showed that PTUPB treatment for 14 days also significantly reduced BLM-induced lung histological changes and collagen deposition in the lungs (Figure 1(a)). PTUPB significantly decreased Collagen I protein (Figures 1(b) and 1(c)) and the expression of tissue inhibitors of metalloproteinase 1 (*Timp1*) mRNA (Figure 1(d)). At the same time, we found that PTUPB significantly reduced *α*-SMA expression and restored E-cadherin expression in the lungs (Figures 1(e)–1(g)). These results suggest that the reduction of PF by PTUPB is related to the reduction of EMT.

### 3.2. COX-2 and sEH Expression Are Increased in TGF-*β*1-Treated AECs

**Figure 2:**
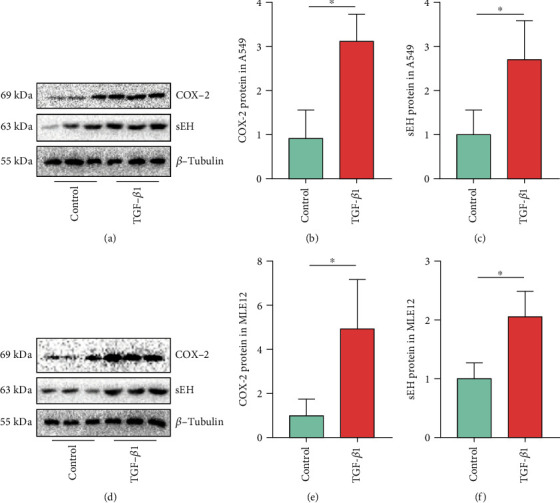
COX-2 and sEH expression are increased in TGF-*β*1-treated AECs. COX-2 and sEH protein expressions in A549 cells (a–c) and MLE12 cells (d–f) were detected using western blot (*n* = 3). The data shown are from a representative experiment with biological triplicates. Data are expressed as the mean ± SD. Differences between the two groups were determined by an unpaired *t*-test. ∗*P* < 0.05.

The protein expressions of COX-2 and sEH were detected in TGF-*β*1-treated A549 and MLE-12 cells. We found that both COX-2 and sEH protein levels were increased in TGF-*β*1-treated A549 (Figures 2(a)–2(c)) and MLE-12 cells (Figures 2(d)–2(f)), indicating that dysregulation of ARA metabolism participates in the development of EMT. These results suggest an important role of COX-2 and sEH dysregulation in the development of EMT.

### 3.3. Prophylactic Treatment of PTUPB Suppresses the TGF-*β*1-Induced EMT in AECs

**Figure 3:**
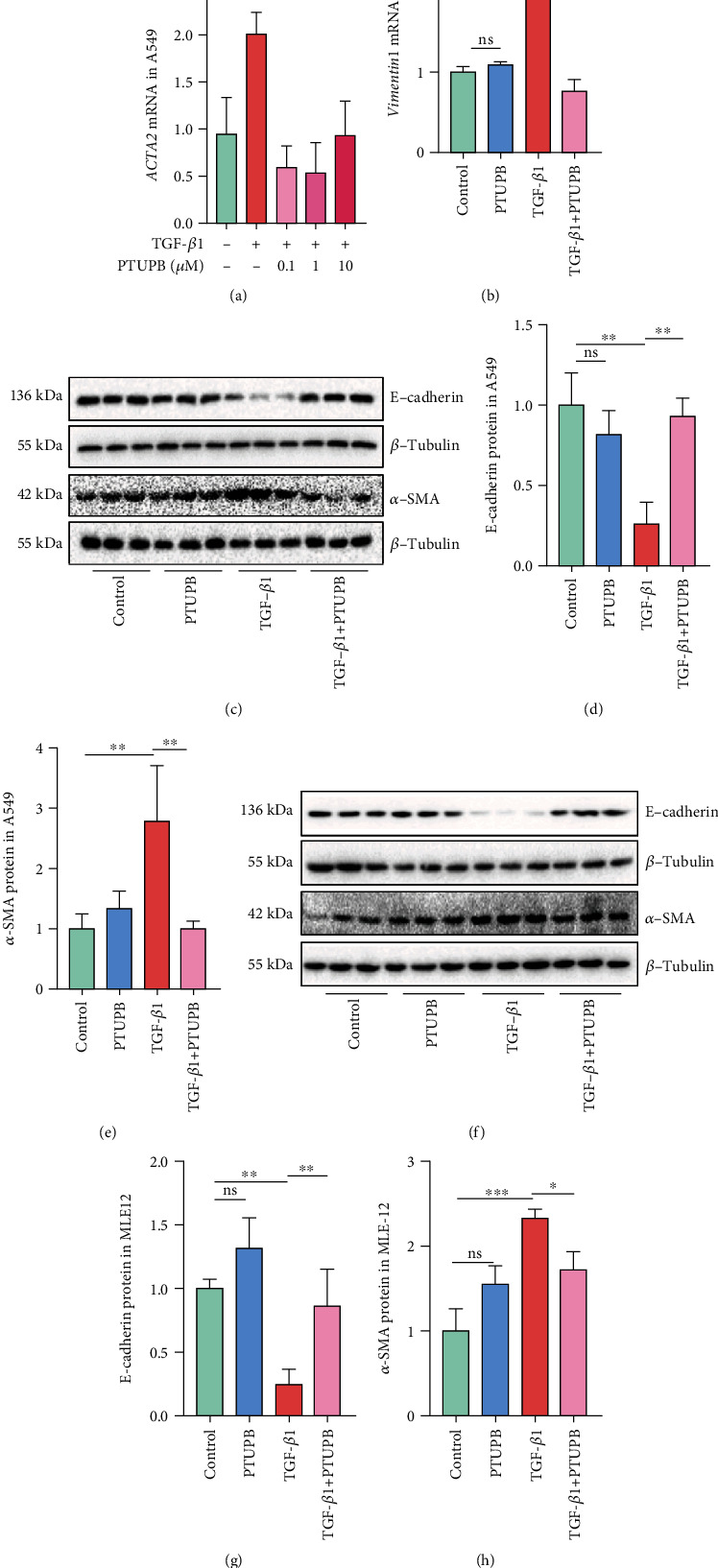
Prophylactic treatment of PTUPB suppresses the TGF-*β*1-induced EMT in A549 and MLE-12 cells. Cells were treated with PTUPB (1 *μ*M) for 1 h before the treatment with TGF-*β*1 (10 ng/mL). The mRNA expressions of *ACTA2* (a) and *Vimentin* (b) in A549 cells were detected by real-time PCR after TGF-*β*1 stimulation for 12 h (*n* = 3). The protein expressions of E-cadherin and *α*-SMA in A549 cells (c–e) and MLE12 cells (f–h) after TGF-*β*1 stimulation for 48 h were measured by western blotting (*n* = 3). The data shown are from a representative experiment with biological triplicates. Data are expressed as the mean ± SD. Differences among multiple groups were performed using ANOVA. Tukey's test was used as a post hoc test to make pairwise comparisons. ∗*P* < 0.05, ∗∗*P* < 0.01, and ∗∗∗*P* < 0.001.

Then, we wondered whether PTUPB suppressed the EMT induced by TGF-*β*1 *in vitro*. We observed that PTUPB alone did not affect the EMT of A549 cells (Figure S1). Further, we found that the treatment with TGF-*β*1 (10 ng/mL) for 12 h significantly increased the mRNA expression of actin alpha 2 (*ACTA2*) (encoding *α*-SMA) and *Vimentin*, indicating the occurrence of EMT, which was effectively suppressed by the pretreatment with PTUPB in A549 cells (Figures 3(a) and 3(b)). We found that PTUPB (1 *μ*M) was the most effective inhibition concentration. In addition, western blotting results showed that the pretreatment with PTUPB (1 *μ*M) reduced *α*-SMA protein expression and restored E-cadherin protein expression induced by TGF-*β*1 (10 ng/mL) (Figures 3(c)–3(h)). Collectively, these results provide strong evidence that PTUPB directly suppresses the EMT induced by TGF-*β*1 in AECs.

### 3.4. Prophylactic Treatment of PTUPB Inhibits the Migration Induced by TGF-*β*1 in A549 Cells

**Figure 4:**
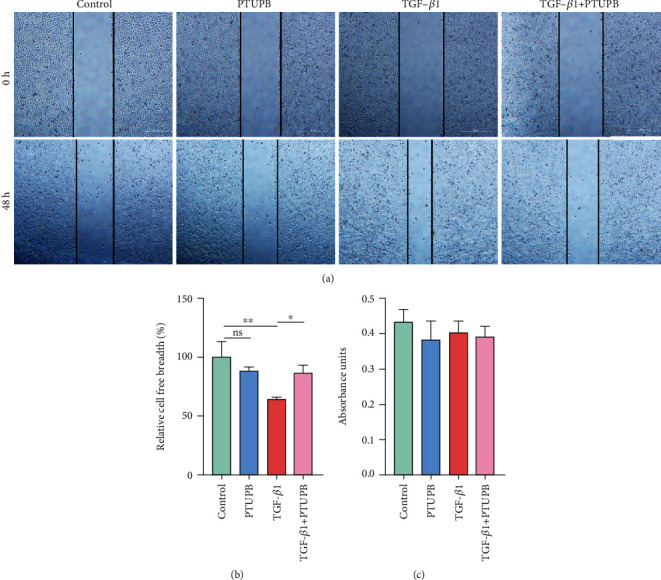
Prophylactic treatment of PTUPB inhibits the migration induced by TGF-*β*1 in A549 cells. Scratch wound healing assay showed that PTUPB (1 *μ*M) inhibited the migratory ability of the A549 cells under the stimulation of TGF-*β*1 (10 ng/mL) ((a–b) *n* = 3, bar =500 px). PTUPB treatment did not affect the proliferation of A549 under low-serum conditions ((c) *n* = 5). The data shown are from a representative experiment with biological triplicates. Data are expressed as the mean ± SD. Differences among multiple groups were performed using ANOVA. Tukey's test was used as a post hoc test to make pairwise comparisons. ∗*P* < 0.05 and ∗∗*P* < 0.01.

We further investigated the effect of PTUPB on TGF-*β*1-induced cell migration with the scratch wound-healing assay. The results showed that TGF-*β*1 treatment (10 ng/mL) for 48 h significantly promoted the migration of A549 cells. PTUPB could significantly reduce this effect (Figures 4(a) and 4(b)). In order to confirm that PTUPB inhibits cell migration but not cell proliferation, we further evaluated proliferation with CCK-8. Results showed that this effect did not attribute to the alteration of cell proliferation (Figure 4(c)). Taken together, these results indicate that PTUPB suppresses cell migration by inhibiting EMT in AECs.

### 3.5. Prophylactic Treatment of PTUPB Inhibits the Collagen Synthesis Induced by TGF-*β*1 in AECs

**Figure 5:**
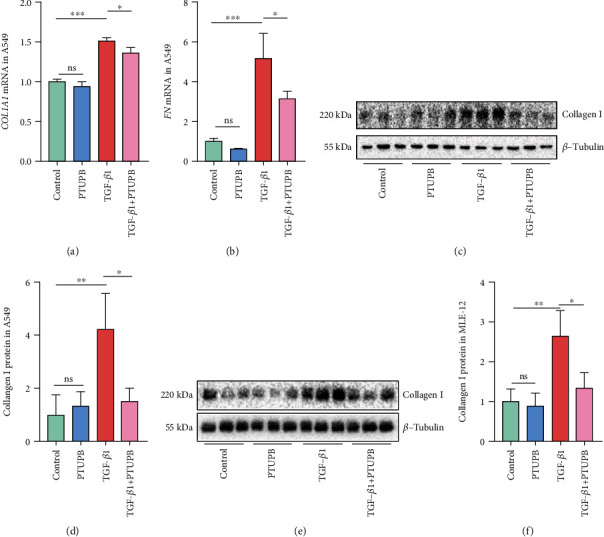
Prophylactic treatment of PTUPB inhibits the collagen synthesis induced by TGF-*β*1 in AECs. Cells were treated with TGF-*β*1 (10 ng/mL) for 24 h present or absent the pretreatment of PTUPB (1 *μM*) for 1 h. The mRNA expressions of *COL1A1* (a) and *FN* (b) in A549 cells were detected by real-time PCR (*n* = 3). The protein expressions of Collagen I in A549 cells (c–d) and MLE-12 cells (e–f) were measured by western blotting after TGF-*β*1 stimulation for 48 h (*n* = 3). The data shown are from a representative experiment with biological triplicates. Data are expressed as the mean ± SD. Differences among multiple groups were performed using ANOVA. Tukey's test was used as a post hoc test to make pairwise comparisons. ∗*P* < 0.05, ∗∗*P* < 0.01, and ∗∗∗*P* < 0.001.

The collagen synthesis can directly reflect the severity of PF. We found that the gene expression of *COL1A1* and fibronectin (*FN*) was significantly increased in A549 cells stimulated by TGF-*β*1, which was effectively suppressed by the pretreatment with PTUPB (Figures 5(a) and 5(b)). TGF-*β*1 treatment also induced the increase of protein expression of Collagen I in A549 cells and MLE-12 cells (Figures 5(c)–5(f)). Pretreatment with PTUPB restored these changes induced by TGF-*β*1. Altogether, these results indicate that PTUPB inhibits the TGF-*β*1-induced collagen synthesis in AECs.

### 3.6. Prophylactic Treatment of PTUPB Disrupts the TGF-*β*1-Smad2/3 Signaling Pathway in AECs

**Figure 6:**
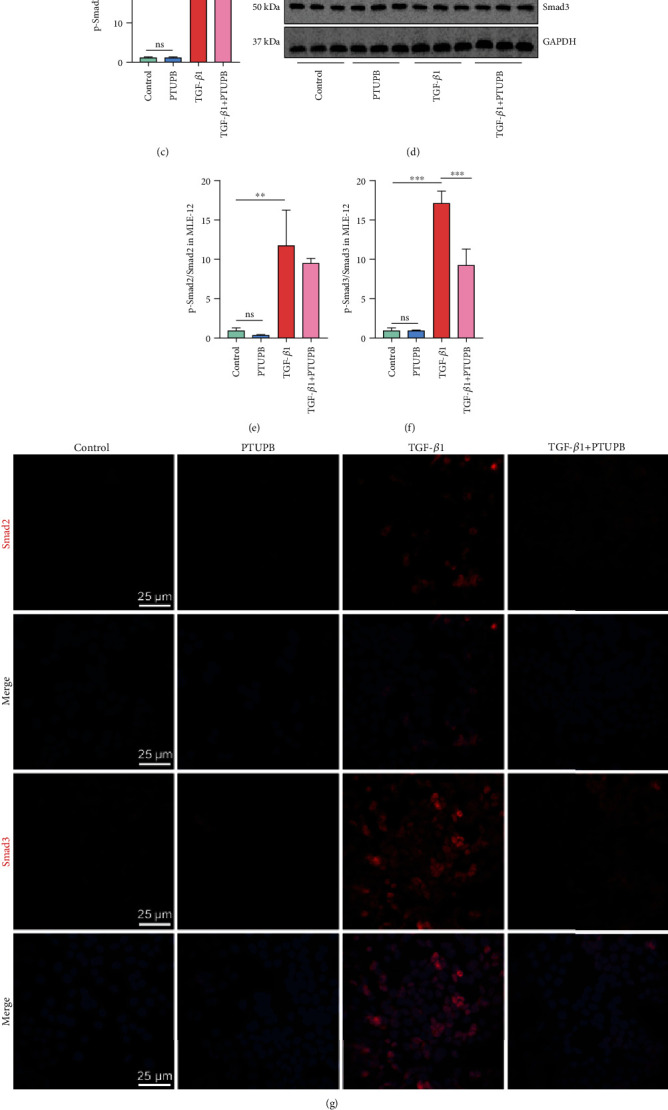
Prophylactic treatment of PTUPB disrupts the TGF-*β*1-Smad2/3 signaling pathway in AECs. Cells were treated with PTUPB (1 *μ*M) for 1 h before the treatment with TGF-*β*1 (10 ng/mL). Thirty minutes after the TGF-*β*1 administration, the levels of p-Smad2 and p-Smad3 in A549 cells ((a–c) *n* = 3) and MLE12 cells ((d–f) *n* = 3) were detected by western blotting. Forty-eight hours after the TGF-*β*1 administration, the fluorescence intensity of Smad2 and Smad3 was detected by immunofluorescence ((g) bar = 25 *μ*m). Twelve hours after the TGF-*β*1 administration, the mRNA expressions of *ZEB1* and *SNAIL1* in A549 cells were detected by real-time PCR ((h–i) *n* = 3). The data shown are from a representative experiment with biological triplicates. Data are expressed as the mean ± SD. Differences among multiple groups were performed using ANOVA. Tukey's test was used as a post hoc test to make pairwise comparisons. ∗*P* < 0.05, ∗∗*P* < 0.01, and ∗∗∗*P* < 0.001.

To elucidate the mechanism of PTUPB on EMT, we focused on the downstream signaling pathways of TGF-*β*1, including Smad, MAPK, and PI3K signaling pathways. We found that PTUPB had no significant effect on MAPK and PI3K signaling pathways after TGF-*β*1 activation (Figure S2). However, after PTUPB pretreatment, TGF-*β*1-induced phosphorylation of Smad2 and Smad3 in A549 cells was significantly reduced (Figures 6(a)–6(c)). Meanwhile, PTUPB was also observed to reduce the phosphorylation of Smad3 in MLE12 cells induced by TGF-*β*1 (Figures 6(d)–6(f)). At this time, the total protein of Smad2/3 in MLE12 cells and A549 cells did not change (Figure S3). Furthermore, immunofluorescence was used to observe that PTUPB reduced nuclear translocation of Smad2 and Smad3 in TGF-*β*1-stimulated MLE12 cells (Figure 6(g)). Then, we found that treatment with PTUPB suppressed the gene expression of the downstream targets of TGF-*β*1-Smad2/3 signaling, including *ZEB1* and *SNAIL1* (Figures 6(h) and 6(i)). These data indicate that PTUPB blocks the TGF-*β*1 signaling pathway through the inhibition of TGF-*β*1-Smad2/3 activation in AECs.

### 3.7. Prophylactic Treatment of PTUPB Restores Nrf2-Dependent Antioxidant Pathways in TGF-*β*1-Induced AECs

**Figure 7:**
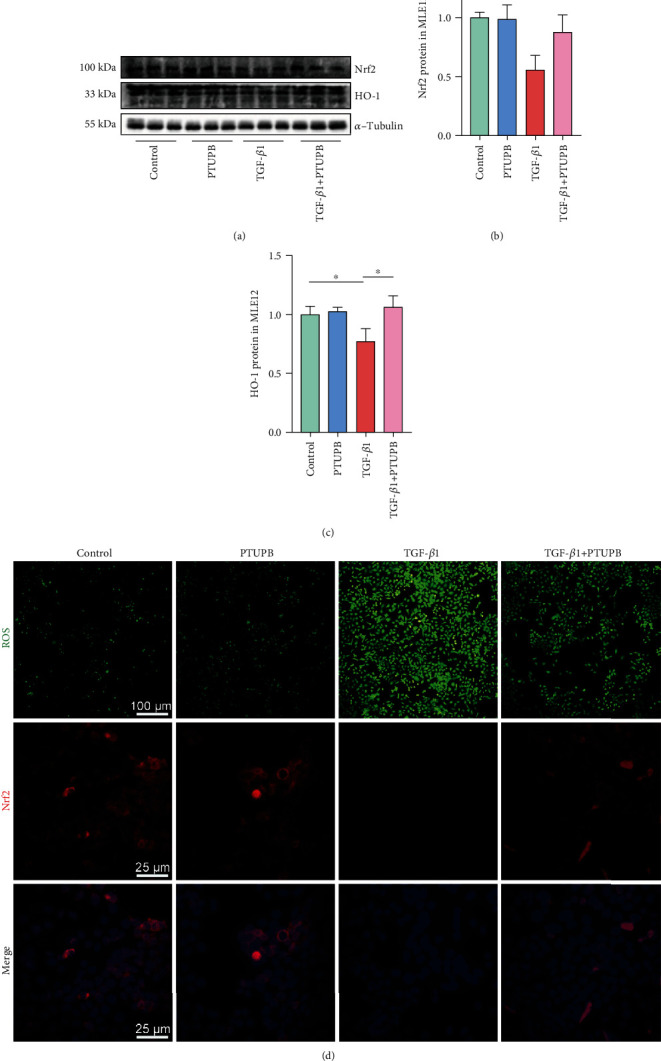
Prophylactic treatment of PTUPB restores Nrf2-dependent antioxidant pathways in TGF-*β*1-treated AECs. Cells were treated with PTUPB (1 *μ*M) for 1 h before the treatment with TGF-*β*1 (10 ng/mL). The protein expressions of Nrf2 and HO-1 in MLE12 cells (a–c) after TGF-*β*1 stimulation for 48 h were measured by western blotting (*n* = 3). The ROS in MLE12 cells after TGF-*β*1 stimulation for 48 h were detected by a ROS kit ((d) bar =100 *μ*m). The fluorescence intensity of Nrf2 was detected by immunofluorescence ((d) bar = 25 *μ*m). The data shown are from a representative experiment with biological triplicates. Data are expressed as the mean ± SD. Differences among multiple groups were performed using ANOVA. Tukey's test was used as a post hoc test to make pairwise comparisons. ∗*P* < 0.05 and ∗∗*P* < 0.01.

TGF-*β*1-induced down-regulation of Nrf2 protein expression and nuclear translocation in MLE12 cells and PTUPB could restore Nrf2 protein expression and nuclear translocation (Figures 7(a), 7(b), and 7(d)). Meanwhile, PTUPB up-regulated HO-1 protein expression downstream of Nrf2 (Figures 7(a) and 7(c)). Activation of Nrf2 is beneficial to ROS elimination. As expected, TGF-*β*1 increased ROS in MLE12 cells, and PTUPB decreased ROS content (Figure 7(d)). These results suggest that PTUPB could enhance the Nrf2 signaling pathway in AECs.

### 3.8. Inhibition of Nrf2 Attenuates PTUPB Regulation of TGF-*β*1/Smad Signaling

**Figure 8:**
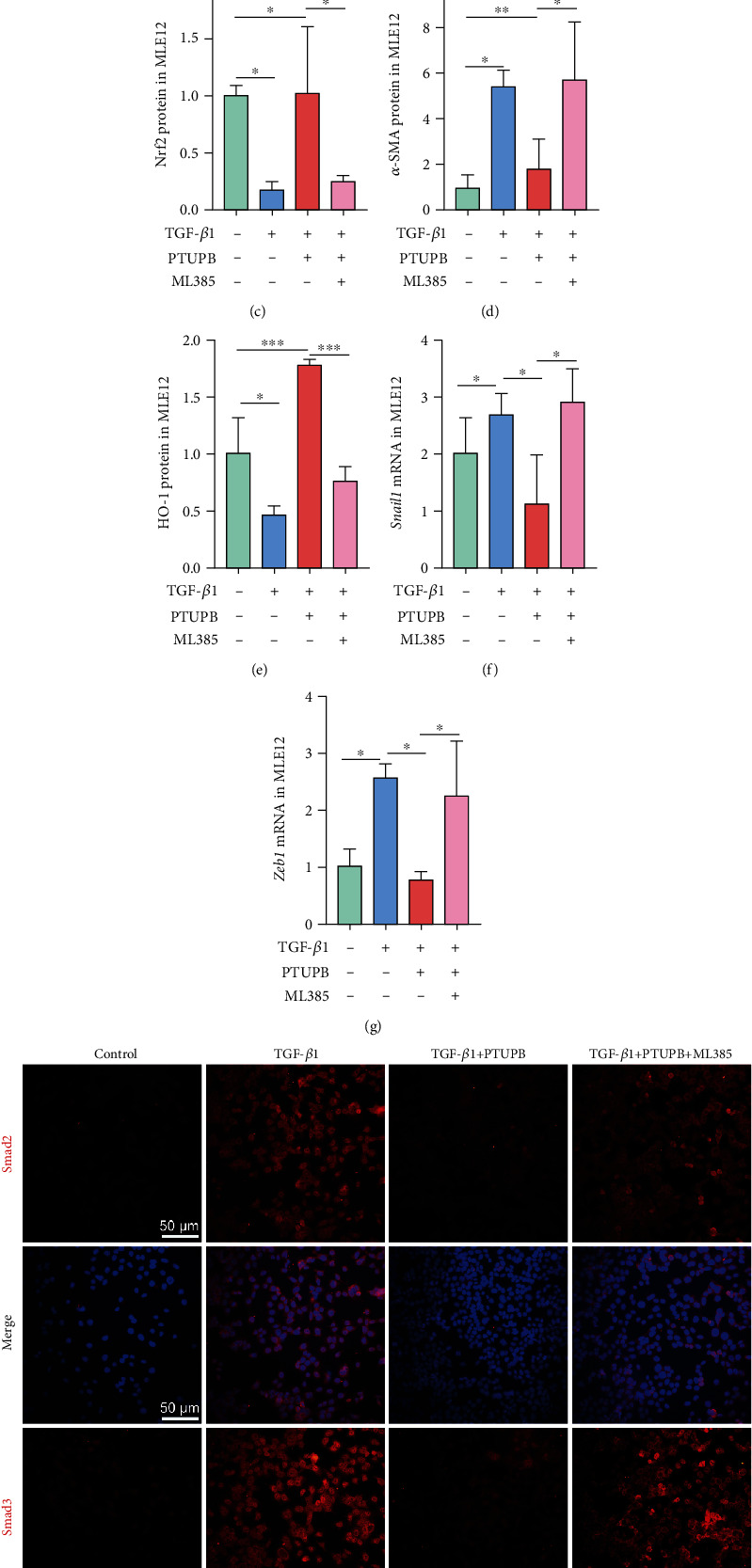
Inhibition of Nrf2 attenuates PTUPB regulation of TGF-*β*1/Smad signaling. Cells were treated with PTUPB (1 *μ*M) and ML385 (5 *μ*M) for 1 h before the treatment with TGF-*β*1 (10 ng/mL). The protein expressions of Nrf2, HO-1, E-cadherin, and *α*-SMA in MLE12 cells (a–e) after TGF-*β*1 stimulation for 48 h were measured by western blotting (*n* = 3). Twelve hours after the TGF-*β*1 administration, the mRNA expressions of *Zeb1* and *Snail1* in MLE12 cells were detected by real-time PCR ((f–g) *n* = 3). Forty-eight hours after the TGF-*β*1 administration, the fluorescence intensity of Smad2 and Smad3 was detected by immunofluorescence ((h) bar =50 *μ*m). The data shown are from a representative experiment with biological triplicates. Data are expressed as the mean ± SD. Differences among multiple groups were performed using ANOVA. Tukey's test was used as a post hoc test to make pairwise comparisons. ∗*P* < 0.05, ∗∗*P* < 0.01, and ∗∗∗*P* < 0.001.

In MLE12 cells, inhibition of EMT by PTUPB was eliminated by blocking the Nrf2-HO-1 signaling pathway (Figures 8(a)–8(e)). PTUPB-mediated nuclear translocations of Smad2 and Smad3 were also reduced with Nrf2 inhibition (Figure 8(h)). Similarly, PTUPB-inhibited transcription of Smad2/3-downstream genes was also abolished (Figures 8(f) and 8(g)). These results suggest that activation of Nrf2 plays an important role in the regulatory effects of PTUPB on the TGF-*β*1/Smad axis.

## 4. Discussion

The transition of AECs into mesenchymal cells has been reported to cause and/or aggravate PF [6]. In this study, the direct effects of PTUPB on the TGF-*β*1-induced EMT were investigated. We found that PTUPB restored the phenotype changes, reduced the migration ability, and inhibited the collagen synthesis of TGF-*β*1-stimulated AECs by disrupting the TGF-*β*1-Smad2/3 pathway. We demonstrate for the first time that PTUPB blocks TGF-*β*1-induced EMT in AECs by *inhibiting* the TGF-*β*1-Smad2/3 signaling pathway. We found that PTUPB restored phenotypic changes, reduced migration ability, and inhibited collagen synthesis of TGF-*β*1-stimulated AECs. We demonstrated for the first time that PTUPB blocks EMT of AECs by up-regulating Nrf2 and inhibiting the TGF-*β*1-Smad2/3 signaling pathway.

ARA is one of the most abundant polyunsaturated fatty acids in the body [39]. ARA is involved in a variety of biological processes, such as angiogenesis, cell migration, and apoptosis [40]. It has been found that inhibiting sEH could increase endogenous EETs content and reduce the EMT process [29, 41]. 14,15-EET and its synthetic analog EET-A could decrease the expression of the EMT inducer factors, ZEB1 and Snail1, prevent the decrease of E-cadherin, and reduce the expression of mesenchymal/myofibroblast markers in the UUO model [29]. However, another ARA pathway, COX-2 metabolism, promotes EMT. COX-2 inhibitor-induced EMT reversal with restored E-cadherin expression has been observed in several cancer cells [42, 43]. The COX-2 metabolite PGJ2 induces EMT by up-regulating the expression of snails [44]. It can be seen that different metabolites of ARA play different roles in the process of EMT. We found that the protein expression of sEH and COX-2 increased significantly during the TGF-*β*1-induced EMT process, manifested by the CYP/COX-2 metabolism disorder in ARA.

Studies have found a common phenomenon in the three metabolic pathways of ARA: inhibition of any one of these pathways may shunt ARA to the other pathway, thereby reducing efficacy and causing adverse reactions [45–47]. For example, NSAIDs may have anti-inflammatory effects by inhibiting COX, but their side effects may increase the risk of stroke and kidney failure [48]. At the same time, selective inhibition of COX-2 reduces the levels of endothelin PGI2 and the platelet aggregator TXA2, which increases the risk of cardiovascular disease [46]. Therefore, the development of bimolecular inhibitors targeting ARA metabolism has become increasingly important. It has long been found that drugs targeting a single molecule can produce other toxicity and drug resistance, while drugs targeting multiple molecules are less likely to develop resistance and have better therapeutic effects [49]. PTUPB is a novel COX-2 and sEH dual inhibitor [31], and we demonstrated that PTUPB could suppress PF [30], acute lung injury [33], nonalcoholic fatty liver disease [34], and sepsis [35]. However, the direct effects of PTUPB on TGF-*β*1-induced EMT in AECs are unknown. In the present study, PTUPB significantly improved E-cadherin expression, decreased *α*-SMA expression, and reduced excessive extracellular matrix deposition in BLM-treated mice. TIMPs serve an important role in controlling tissue organization and fibrosis following injury [50]. We found that PTUPB decreased the expression of *Timp1* mRNA in BLM-treated PF mice lung tissue, which may be one of the reasons for decreased collagen synthesis.

Further, *in vitro* EMT models of MLE-12 and A549 cells were induced by exogenous TGF-*β*1. We found that PTUPB attenuated TGF-*β*1-induced the acquisition of mesenchymal markers (such as *α*-SMA), prevented TGF-*β*1-induced the loss of epithelial markers (such as E-cadherin), decreased TGF-*β*1-induced the enhancement of migration ability, and reduced TGF-*β*1-induced the accumulation of collagen synthesis. These results suggest that regulating COX-2/CYP metabolism in AECs alleviates TGF-*β*1-induced EMT. Our results support the hypothesis that inhibition of COX-2/sEH by PTUPB potently inhibits the progression of EMT. In short, our findings indicate that a COX-2 and sEH dual inhibitor show a pivotal therapeutic potential for EMT.

ROS plays an important role in the process of EMT, and TGF-*β*1-induced EMT can be inhibited by interfering with related upstream molecular events or by treating cells with antioxidants to block ROS production [51, 52]. These studies indicate that ROS production is an important signal for EMT initiation. It has been found that restoring intracellular antioxidant signaling pathways can reduce TGF-*β*1-induced EMT. For example, piperine enhances the Nrf2 antioxidant cascade, reduces TGF-*β*1-induced ROS accumulation, and eliminates EMT in AML-12 hepatocytes [14]. Our data show that PTUPB restored Nrf2 protein expression and nuclear translocation in TGF-*β*1-stimulated MLE12 cells, while reducing TGF-*β*1-induced intracellular ROS levels. In addition, we unveiled that inhibition of Nrf2 abrogated the protective activity of PTUPB against TGF-*β*1. Thus, it is reasonable to speculate that targeted activation of Nrf2 is a pivotal contributor to the lung-protective activity of PTUPB.

TGF-*β*1-activated Smads play an important role in the process of EMT [53]. The combination of activated Smad2 or Smad3 and Smad4 can transcriptionally regulate EMT, while blocking the expression of Smad2 or Smad3 can reduce TGF-*β*1-induced EMT [54]. TGF-*β*1 activates T*β*RI by acting on the receptor complex and directly phosphorylates the C-terminal of Smad2 and Smad3. After phosphorylation, Smad2, Smad3, and Smad4 form trimers, which are transported to the nucleus, bind to DNA-binding transcription factors, and cooperatively regulate the transcription of target genes [53]. Our study found that PTUPB significantly reduced TGF-*β*1-induced phosphorylation of Smad2 and Smad3 in A549. Meanwhile, PTUPB also reduced the phosphorylation level of Smad3 induced by TGF-*β*1 in MLE12 and tended to decrease the phosphorylation level of Smad2 induced by TGF-*β*1 in MLE12. From the multiple of Smad2/3 phosphorylation change, we believe that PTUPB mainly inhibited the phosphorylation level of Smad3 in AECs. It was further found that PTUPB decreased the expression of *ZEB1* mRNA and *SNAIL1* mRNA downstream of the TGF-*β*1-Smad signaling pathway. These data indicate that PTUPB could inhibit activation of the TGF-*β*1-Smad2/3 pathway, therefore suppressing TGF-*β*1-induced EMT. Moreover, we also unveiled that in MLE12 cells, inhibition of Nrf2 crippled the regulatory effects of PTUPB on TGF-*β*1/Smad signaling. This finding suggests that activation of Nrf2 is an important upstream event that explains PTUPB-mediated modulation of intracellular TGF-*β*1/Smad pathways.

## 5. Conclusion

**Figure 9:**
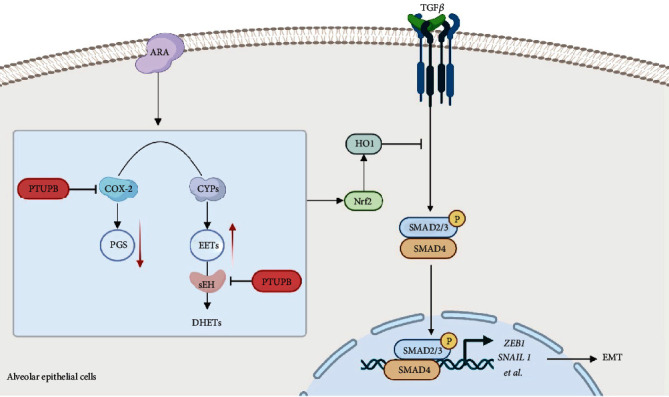
Schematic illustration. A COX-2/sEH dual inhibitor PTUPB inhibits EMT induced by TGF-*β*1 in AECs.

In summary, our findings demonstrate that the disorder in the COX-2/CYP metabolism of ARA plays a role in TGF-*β*1-induced EMT. PTUPB inhibits the activation of the TGF-*β*1-Smad2/3 pathway through the Nrf2 antioxidant cascade, thus inhibiting EMT in AECs (Figure 9). This study might promote the application of PTUPB in PF treatment.

## Figures and Tables

**Table 1:** Sequences of specific primers were used in this study.

Gene	Forward primer (5′-3′)	Reverse primer (5′-3′)
*m-Snail1*	GTCCAGCTGTAACCATGCCT	TGTCACCAGGACAAATGGGG
*m-Zeb1*	GCTGGCAAGACAACGTGAAAG	GCCTCAGGATAAATGACGGC
*m-β-actin*	TTCCAGCCTTCCTTCTTG	GGAGCCAGAGCAGTAATC
*h-TIMP1*	AGAGTGTCTGCGGATACTTCC	CCAACAGTGTAGGTCTTGGTG
*h-MMP9*	TGTACCGCTATGGTTACACTCG	GGCAGGGACAGTTGCTTCT
*h-CDH1*	GCTGGACCGAGAGAGTTTCC	CAAAATCCAAGCCCGTGGTG
*h-ACTA2*	AAAGCAAGTCCTCCAGCGTT	TTAGTCCCGGGGATAGGCAA
*h-Vimentin*	GTCCGCACATTCGAGCAAAG	TGAGGGCTCCTAGCGGTTTA
*h-COL1A1*	CCTGGATGCCATCAAAGTCT	AATCCATCGGTCATGCTCTC
*h-FN*	AAACCAATTCTTGGAGCAGG	CCATAAAGGGCAACCAAGAG
*h-ZEB1*	TTACACCTTTGCATACAGAACCC	TTTACGATTACACCCAGACTGC
*h-SNAIL1*	CTAGGCCCTGGCTGCTACAA	CCTGGCACTGGTACTTCTTGA
*h-GAPDH*	AATTCCATGGCACCGTCAAG	TGGACTCCACGACGTACTCA

## Data Availability

The datasets generated during and/or analyzed during the current study are available from the corresponding author on reasonable request.

## Abbreviations

ACTA2: Actin alpha 2
AECs: Alveolar epithelial cells
ARA: Arachidonic acid
BLM: Bleomycin
CDH1: Cadherin1
COL1A1: Collagen type I alpha 1
COL1A3: Collagen type I alpha 3
COX: Cyclooxygenase
CYP: Cytochrome P450 oxidase
EMT: Epithelial-mesenchymal transition
EETs: Epoxyeicosatrienoic acids
FN: Fibronectin
HO-1: Heme oxygenase-1
LTs: Leukotrienes
LOX: Lipoxygenase
Nrf2: Nuclear factor erythroid 2-related factor-2
PGs: Prostaglandins
PF: Pulmonary fibrosis
ROS: Reactive oxygen species
sEH: Soluble epoxide hydrolase
TGF-*β*1: Transforming growth factor
TIMP1: Tissue inhibitor of metalloproteinase1
UUO: Unilateral ureteral obstruction
*α*-SMA: *α*-Smooth muscle actin
PTUPB: 4-(5-phenyl-3-{3-[3-(4-trifluoromethylphenyl)-ureido]-propyl}-pyrazol-1-yl)-benzenesulfonamide
ZEB: Zinc-finger E-box binding.

## Additional Points

*Authors declare*. The preprint has previously been published [55].

## Supplementary Materials

Supplementary Materials Figure S1. Effects of different concentrations of PTUPB on mRNA expression of TIMP1, MMP9, and CDH1 in A549 cells. Figure S2. Prophylactic treatments of PTUPB have no effect on the PI3K and MAPK signaling pathways of AECs. Figure S3. Prophylactic treatment of PTUPB does not affect total Smads protein in AECs.
