# Supplementary material for: COX-2/sEH Dual Inhibitor PTUPB Attenuates Epithelial-Mesenchymal Transformation of Alveolar Epithelial Cells via Nrf2-Mediated Inhibition of TGF-β1/Smad Signaling
Source: Oxid Med Cell Longev. 2022 Apr 25;2022:5759626. doi: 10.1155/2022/5759626 (PMC9060975; doi:10.1155/2022/5759626)
Supplement: Supplementary Materials — Figure S1. Effects of different concentrations of PTUPB on mRNA expression of TIMP1, MMP9, and CDH1 in A549 cells. Figure S2. Prophylactic treatments of PTUPB have no effect on the PI3K and MAPK signaling pathways of AECs. Figure S3. Prophylactic treatment of PTUPB does not affect total Smads protein in AECs. [file 5759626.f1.docx]

**Supplementary Figures and Figure legends**


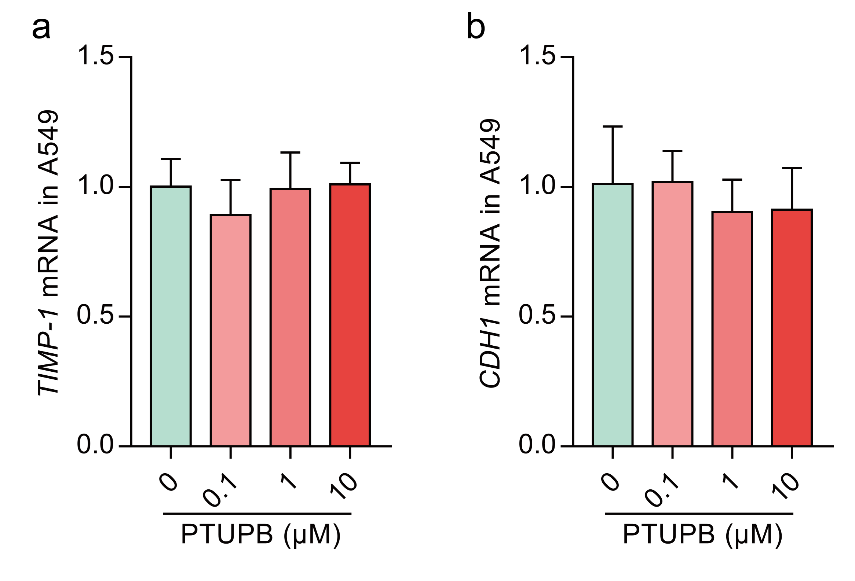


c d


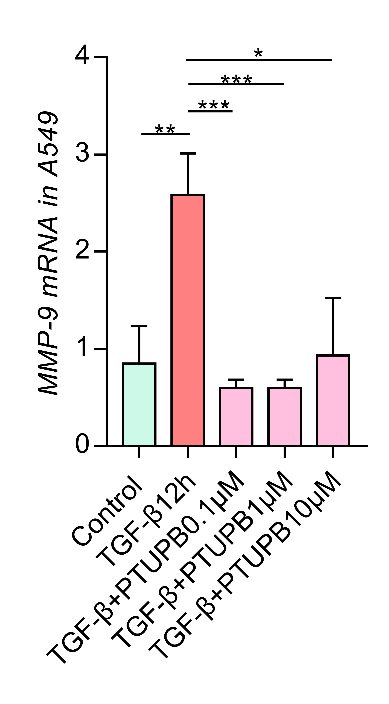

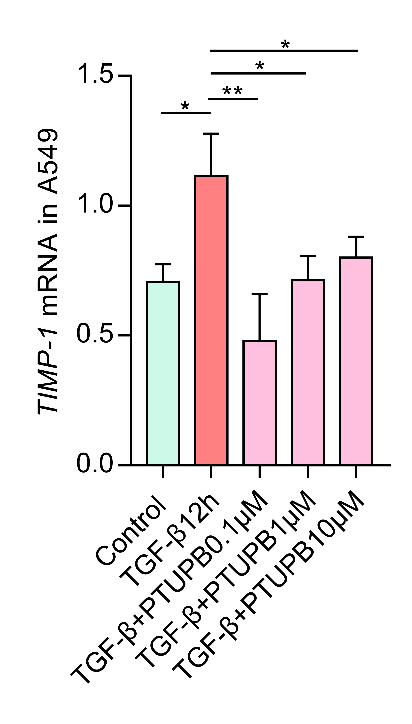


**Fig. S1. Effects of different concentrations of PTUPB on mRNA expression of *TIMP1*, *MMP9,* and *CDH1* in A549 cells.** A549 cells were treated with serial concentrations of PTUPB (0, 0.1, 1, and 10 μM) for 12 h. The *TIMP1* mRNA (a) and *CDH1* mRNA (b) were detected by real-time PCR (*n*=3). Cells were treated with PTUPB (0.1, 1, 10 μM) for 1 h before the treatment with TGF-β1 (10 ng/mL). The mRNA expressions of *MMP9* (c) and *TIMP1* (d) in A549 cells were detected by real-time PCR after TGF-β1 stimulation for 12 h (n=3). Data are expressed as the mean ± SD. Differences between two groups were determined by unpaired *t*-test. Differences among multiple groups were performed using ANOVA. Tukey’s test was used as a post hoc test to make pairwise comparisons. * *P* < 0.05, ** *P* < 0.01 and *** *P* < 0.001.


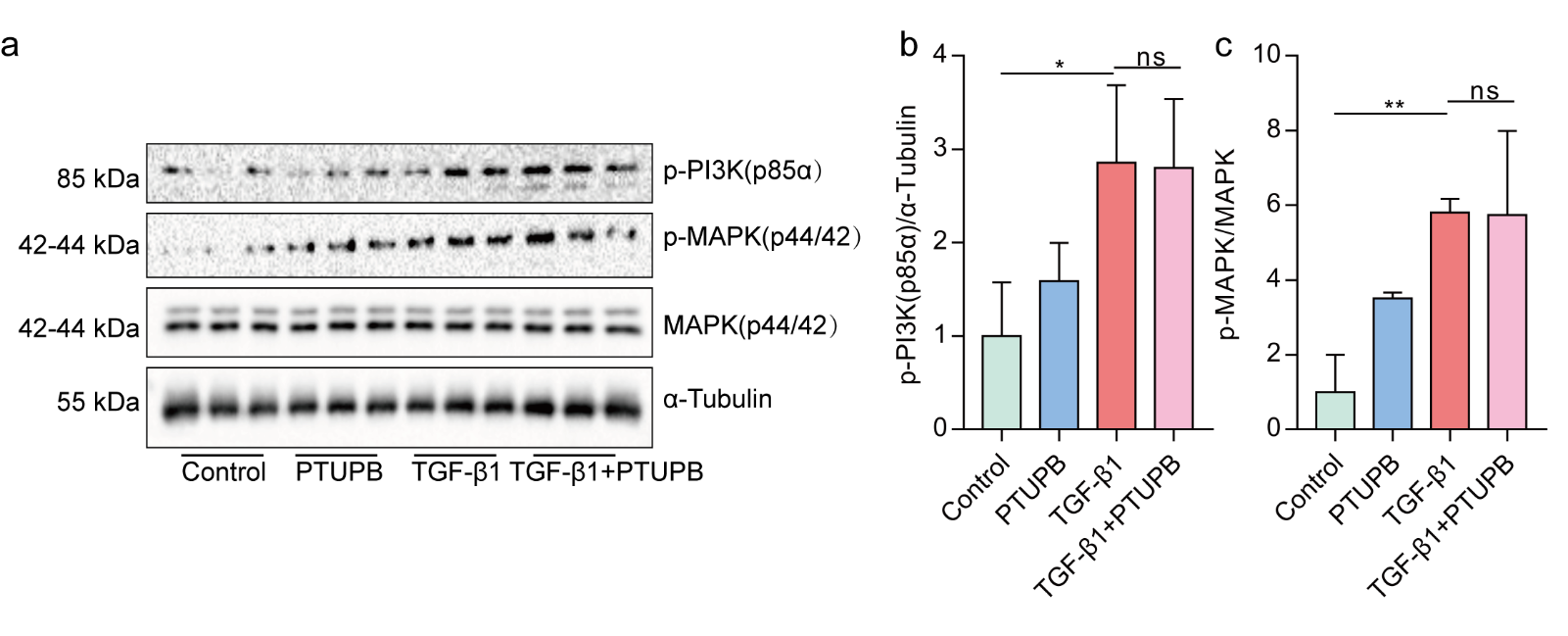


**Fig. S2. Prophylactic treatment of PTUPB has no effect on the PI3K and MAPK signaling pathways of AECs.** Cells were treated with PTUPB (1 μM) for 1 h before the treatment with TGF-β1 (10 ng/mL). Thirty minutes after the TGF-β1 administration, the protein expressions of p-PI3K (p85), p-MAPK (p42/44), and MAPK (p42/44) in MLE12 cells (a-c, *n* = 3) were detected by western blotting. Data are expressed as the mean ± SD. Differences among multiple groups were performed using ANOVA. Tukey’s test was used as a post hoc test to make pairwise comparisons. * *P* < 0.05


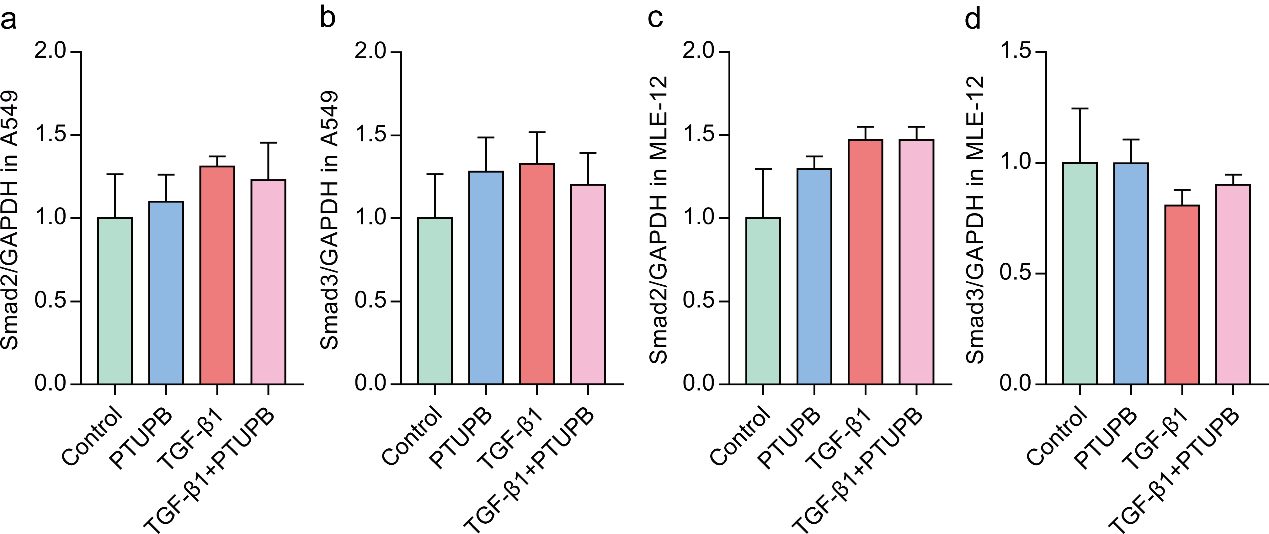


**Fig. S3.** **Prophylactic treatment of PTUPB has no effect on total Smads protein in AECs.** Cells were treated with PTUPB (1 μM) for 1 h before the treatment with TGF-β1 (10 ng/mL). Thirty minutes after the TGF-β1 administration, the protein expressions of Smad2 and Smad3 in A549 cells (a-b, *n* = 3) and MLE12 cells (c-d, *n* = 3) were detected by western blotting. Data are expressed as the mean ± SD. Differences among multiple groups were performed using ANOVA. Tukey’s test was used as a post hoc test to make pairwise comparisons.
